# Continuous-Flow Cell Dipping and Medium Exchange in a Microdevice using Dielectrophoresis

**DOI:** 10.3390/mi9050223

**Published:** 2018-05-08

**Authors:** Falah Alhammadi, Waqas Waheed, Bashar El-Khasawneh, Anas Alazzam

**Affiliations:** Mechanical Engineering Department, Khalifa University of Science and Technology, Abu Dhabi 127788, UAE; 100031581@ku.ac.ae (F.A.); waqas.waheed@ku.ac.ae (W.W.); bashar.khasawneh@ku.ac.ae (B.E.-K.)

**Keywords:** dipping, medium exchange, dielectrophoresis, microfluidics, microchannel, parametric studies, blood cells

## Abstract

Medium exchange is the process of changing the suspension medium of cells/particles, and has applications in washing, surface modifications, nutrient replenishment, or simply changing the environment of the target entities. Dipping involves diverting the path of target cells in the carrying fluid to immerse them in another fluid for a short duration, and pushing them again into the original medium. In this paper, a simple microfluidic platform is introduced that employs dielectrophoresis to achieve medium exchange and dipping of micro-objects in a continuous manner. The essential feature of the platform is a microchannel that includes two arrays of microelectrodes that partly enter the bottom surface from both sides. In the first step, numerous finite element-based parametric studies are carried out to obtain the optimized geometrical and operational parameters ensuring successful dipping and medium exchange processes. The results of those studies are utilized to fabricate the platform using standard photolithography techniques. The electrodes are patterned on a glass substrate, while the channel, made out of polydimethylsiloxane, is bonded on top of the glass. Trajectories of blood cells from numerical studies and experimentations are reported, and both results exhibited close agreement.

## 1. Introduction

Sample preparation is the first and essential step in any kind of analytical system in the fields of biotechnology, food processing, medical diagnostics, and microassay systems. Often, the success of these analyses relies on the accuracy and the quality of the sample being used. The conventional sample preparation procedures are tedious, require highly skilled labor, and are extremely time consuming. Furthermore, the conventional cell analysis techniques are based on extracting the averaged properties of a population of cells that are prone to inaccuracies because of the heterogeneous gene expression and large variations in the behavior and physical properties, even in the same type of cells. The problem gets more complicated when detections/operations of rare cells, for instance, detection and isolation of circulating cancer cells from blood samples, are involved. Microfluidics has emerged as a promising field with the potential to improve traditional sample preparation methods, diagnostics, and prognostic techniques [1]. The ability of single cell manipulation has been the driving factor in the development of microfluidic lab-on-chip platforms, to gain an insight into cellular behavior in response to controlled stimuli. These single-cell operations have enabled accurate electrical and mechanical characterization of individual cells [2,3], and are increasingly applied in the fields of disease detection, immunology, plant biology, environmental monitoring, and medical diagnostics [4]. Other great advantages offered by microfluidics are small sample usage, cost-effectiveness, fast response, portability, less wastage of material, ease of integration, and biocompatibility.

The target micro-objects can be manipulated in two common ways: either trapping them in physical or virtual cages, or allowing them to move in a continuous-flow method. The former method is more cumbersome, and requires a special protocol for successful operation. The continuous-flow based method, on the contrary, offers the advantages of being less labor-intensive, has high throughput, and reduces the time of operation significantly. Continuous cell manipulation methods can be further classified into active and passive methods. Active approaches employ an external force for continuous manipulation of cells into a designated position in a microchannel. In passive continuous-flow methods, the cells are influenced by hydrodynamic forces and the geometry of the microchannel.

Various innovative techniques have been used efficiently for effective and controlled manipulation of micro-objects. Magnetic techniques have been utilized to divert the paths of mammalian cells laterally in a ferrofluid solution [5]. Piezoelectric transducers have effectively been used to generate acoustic forces for medium exchange of polystyrene (PS) particles [6]. Other techniques for manipulation of single cells include optical [7], electric [8], and mechanical [9] methods. Among them, the electrical-based technique, called dielectrophoresis (DEP) [10], to perform medium exchange and cell dipping, is introduced in this work. Dielectrophoresis is a popular choice for cell manipulation because of its simplicity, high selectivity, and ease of integration with other microfluidic devices [11]. The non-uniform electric field for DEP force in the microchannel is generated using a variety of electrode configurations, including coplanar configuration [12,13], top and bottom configuration [14], sidewall electrodes [15], and the more recently introduced inherently aligned and customized liquid metal electrodes [16,17].

Medium exchange in microchannels is the process of transporting cells suspended in a medium to another parallel-flowing medium inside the same channel. The target cells are deflected once in the microchannel by applying an external force field. Cell dipping is the process of changing the environment of the target cells twice. The cells are diverted from the first carrying medium to the second one for a short duration, and are then brought back to the original medium [18]. Turbulent mixing of fluids does not take place in microscaled devices because of the extremely slow laminar flows, leading to extremely small Reynolds numbers, ~0.01–1. Mixing by diffusion requires a long channel, and can be easily avoided by using shorter microchannels [19]. The applications of dipping phenomenon are numerous: it can be successfully utilized to exchange the medium of the target particles—such as cells or their constituents—before further processing. The medium can be a buffer solution, either for cell washing or eliminating cellular waste materials. In other instances, the medium may also be a reagent intended for surface functionalization, labeling, or refilling the nutrients of the target species. Since extremely small amounts of the sample and mediums are involved, all the target particles are exposed to the same concentration of the desired medium, decreasing the possibility of functionalization non-uniformity for the given population of particles. Lastly, the techniques can be influential in opening new avenues for on-chip study of cells, particles, proteins, and DNA.

We have introduced a dielectrophoresis-based microdevice to perform cell/particle medium exchange and dipping in a continuous-flow manner. The simple design of the microdevice makes it possible to fabricate the microdevice using standard microfabrication only. Hence, the fabrication of the microdevice is easier, as compared to the devices with top-bottom or three-dimensional vertical electrodes arrangement [15]. Moreover, a higher efficiency in cell dipping and medium exchange is possible, since the current microdevice creates a higher non-uniform electric field as compared to the parallel vertical electrodes. The cells/particles are manipulated based on their sizes, as well as their electrical properties, as evident from Equation (1). The operating frequency and electric properties of both the microparticles and the carrying medium are chosen, to ensure that all microparticles experience the repulsive nDEP force from both sets of electrodes. The microparticles, suspended in a medium, are dipped in the adjacent parallel-flowing medium, by pushing them using an electrode set at the surface of the substrate, and under one of the sidewalls of the microchannel. The dipping time of these microparticles can be changed in a controlled manner by changing the flow velocity, the distance between the two sets of electrodes, and the applied voltage and frequency. As the microparticles traverse through the channel, they are pushed towards the original medium again by the nDEP force from the opposite electrodes set. Hence, a very controlled dipping operation is possible by employing the proposed device. For medium exchange, only a single set of electrodes is utilized to immerse the particles in the second medium; the second set is not powered on. In medium exchange applications, the cells that are introduced are carried into the microchannel through one medium, and leave the channel while being suspended in the second medium. 

## 2. Theory

Dielectrophoresis describes the migration of neutral (but polarizable) particles in the presence of a non-uniform electric field [20]. It is a simple, relatively fast, and noninvasive technique that has been extensively used for successful manipulation [21], characterization [22], and separation of microparticles [23], bacteria [24], yeast [25], cancer cells [26], DNA [27], and other microentities [28,29].

When a dielectric object is placed in an inhomogeneous electrical field, its surface charges are redistributed: equal and opposite charges are separated at its boundary, leading to the generation of an induced dipole moment. The direction of translation of the micro-objects depends on the relative polarizability of the target objects compared to the suspending medium. If the micro-object is more polarizable than the medium, it is pushed towards the higher electric field region, and the phenomenon is known as “positive dielectrophoresis (pDEP)”. In the opposite direction, called negative dielectrophoresis (nDEP), the object translates towards regions of lower field strength. Mathematically, the time-averaged DEP force acting on a spherical object of radius $r_{p}$ suspended in a medium having permittivity $\varepsilon_{m}$ in the presence of a non-uniform electric field, is stated as [30]

(1) $$F_{DEP}=2\pi \varepsilon_{m}r_{p}{}^{3}Re\left[f_{CM}\right]\nabla {\left|E_{rms}\right|}^{2}$$

(2) $$Re\left[f_{CM}\right]=\frac{\varepsilon_{p}^{*}-\varepsilon_{m}^{*}}{\varepsilon_{p}^{*}+2\varepsilon_{m}^{*}}$$

where $\varepsilon_{p}^{*}=\;\varepsilon_{p}\;+\;\frac{\sigma_{p}}{\left(j\omega \right)\;}$ and $\varepsilon_{m}^{*}=\;\varepsilon_{m}\;+\;\frac{\sigma_{m}}{\left(j\omega \right)\;}$ represent the complex dielectric permittivity of the target object and the suspending medium, respectively. The term ∇ is the gradient operator, $E_{rms}$ represents the root-mean-squared value of the electric field, and ω  denotes the angular frequency of the applied signal.

The relative polarizability of the micro-objects is described in Equation (2) by the real component of Clausius–Mossotti factor $\;Re\left[f_{CM}\right]$. The range of $Re\left[f_{CM}\right]$ spans from −0.5 to +1; a negative value represents nDEP, whereas it is positive for the pDEP phenomenon.

Numerous researchers have introduced a variety of microfluidic platforms for accurate cell manipulation and dipping. In some of the devices, an electrical trap or a physical barrier was created inside the microchannel to cage the cells. The trapped cells, such as red blood cells (RBCs), were then exposed to multiple fluids—reagents or washing mediums—that were introduced either serially, or by changing relative flow rates of the fluids in the case of side-by-side flowing mediums in a single channel [31]. Continuous-flow cell dipping devices performed medium exchange and washing/cleaning by introducing two parallel-flowing fluids in the channel. The target cells were then deviated multiple times by DEP forces using diagonally-oriented microelectrodes [31]. A two-staged dipping device was developed by cascading two particle exchangers to functionalize nanoparticles. A particle exchanger consisted of two overlapping channels, with buffer and reagent streams flowing in the channels separately. The nanoparticles introduced in a buffer were diverged to the reagent medium in the first stage, and were pushed back later to the cleaning buffer by lateral DEP force generated by the electrode array on one side of the channel [32]. In order to solve the diffusion mixing issue in the device, an optimized version of the device was introduced that employed a wider channel and a combination of dielectrophoresis and electrophoresis phenomenon by patterning electrodes on both sides, and a shifted array of electrodes inside the microchannel [33]. Another technique, called deterministic lateral displacement, initially introduced as continuous-flow particle/cell separation method, was exploited [34]. Other techniques involve the use of standing bulk [35] or surface acoustic waves [36] to switch the micro-objects to different fluids. Tilted-angle standing surface acoustic wave technology is also reported in the literature to displace the cells for washing purpose [37].

## 3. Device Design and Operation

**Figure 1:**
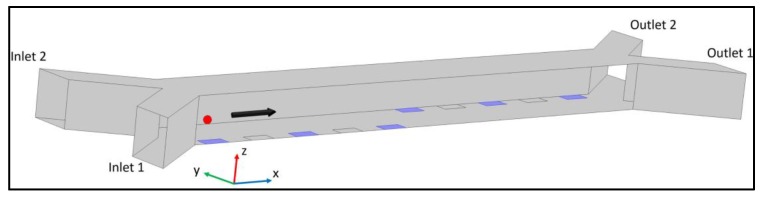
Microchannel design with two arrays of electrodes on opposite sides of the microchannel. Two different fluids enter the microchannel from inlet 1 and inlet 2. The cells (denoted as red object), suspended in a medium, are introduced from inlet 1, and are captured from outlet 1 in the same medium for dipping, and from outlet 2 during medium exchange process. The flow direction is from left to right, as indicated by the arrow.

A schematic diagram of the proposed nDEP-based dipping and medium switching device is shown in Figure 1. The device comprises of a straight microchannel with a set of electrodes with their edges inside the channel at each side of the micro-channel. The device also contains two inlets and two outlets for introducing and discharging both the particles and the two-different mediums. The microparticles are introduced into the channel through the inlet closer to the first set of electrodes. Once the microparticles pass near the first set of electrodes, they will experience a negative dielectrophoretic force that push them away from the electrodes towards the minimum electric field gradient regions. The magnitude of the repulsive force is precisely controlled so that the target objects are immersed in the adjacent fluid stream—the dipping medium. The target microparticles are dipped in the second medium for a predetermined time, and are then pushed back into the original medium by the second set of electrodes using the same principle. The dipping time is mainly controlled by the flow rate of the sample and the distance between the two sets of electrodes.

**Figure 2:**
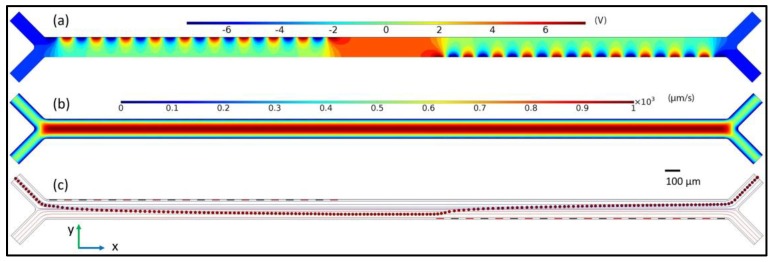
The simulation results from COMSOL Multiphysics 5.2 showing (**a**) the electric potential inside the channel after AC 15 V_p-p_ (Volts) is applied to the electrodes; (**b**) the *x*-component of the velocity field (µm/s) when fluids from two different inlets are introduced into the channel at the same flow rates; and (**c**) the trajectory followed by the microparticles in the microchannel showing successful dipping operation. The red circles represent the particles, whereas the streamlines of both the fluids are represented in blue and red colors. Both the fluids flow from left to right.

In addition to the DEP force, the microparticles are subjected to three other significant forces during their motion inside the microchannel. These forces are sedimentation (the combined effect of gravity and buoyancy forces) and drag forces, as shown in Equation (3). Other effects, such as Brownian force, Joule heating effect, virtual mass effect, particle–particle interaction, particle–wall interactions, and wall repulsion, are assumed negligible, and are neglected [38,39] . The finite element analyses of the microdevice are shown in Figure 2.

(3) $$\left[\begin{array}{c} F_{x}\\ F_{y}\\ F_{z} \end{array}\right]=\;\left[\begin{array}{c} 0\\ 0\\ \left(\rho_{m}-\rho_{p}\right)gv_{p} \end{array}\right]-\;6\pi \mu r_{p}\left[\begin{array}{c} {\left(v_{p}-u\right)}_{x}\\ {\left(v_{p}-u\right)}_{y}\\ {\left(v_{p}-u\right)}_{z} \end{array}\right]+2\pi \varepsilon_{m}r_{p}^{3}Re\left[f_{CM}\right]\left[\begin{array}{c} \frac{\partial }{\partial x}E_{rms}^{2}\\ \frac{\partial }{\partial y}E_{rms}^{2}\\ \frac{\partial }{\partial z}E_{rms}^{2} \end{array}\right]$$

In Equation (3), μ represents fluid viscosity [N·s·m^−2^], and $\rho_{p}$ is the density of the microparticles [kg·m^−3^]. Moreover, the terms u and $v_{p}$ denote the velocity of the fluid medium and that of the particle [m·s^−1^], respectively. Finally, the term g is the acceleration due to gravity [m·s^−2^]. The second term on the right-hand side of Equation (2) represents the drag force experienced by a single microparticle in the fluid. For the case of multiple particles, the shielding effect reduces the drag forces for microparticles that are in close proximity to each other. The magnitude of the reduced drag is obtained by multiplying the drag force with a drag correction factor *D_cor_* given in Equation (4) [38]:

(4) $$D_{cor}\;=\;\frac{1}{1+k_{density\;}\cdot Particle\;Density}$$

The value of $k_{density\;}(\mathrm{density}\;\mathrm{scaling}\;\mathrm{factor}$) in Equation (4) is obtained empirically, and is detailed in [38].

## 4. Microdevice Optimization

In this section, the effect of numerous geometric and operational parameters on particle trajectories is discussed. The parameters studied include channel height, electrode width/gap length, particle diameter, applied voltage, and the flow rate of both mediums. MATLAB and COMSOL Multiphysics^TM^ are used to perform all the parametric studies. Since the two sets of electrodes are identical, the effect of only one set on target objects is studied.

**Figure 3:**
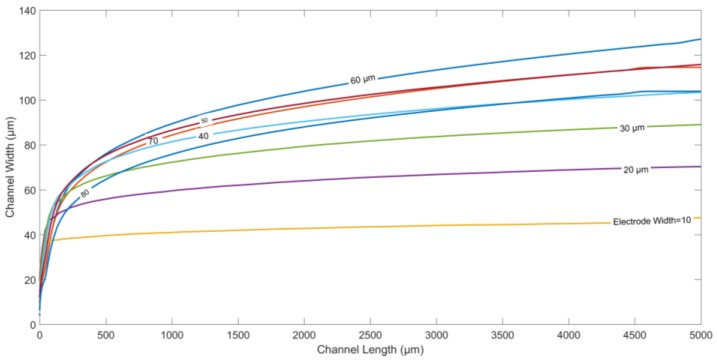
Particle trajectories for different electrode/gap width values calculated using FEM. Applied frequency: 10 kHz. Medium conductivity: 10 mS/m. Medium density: 1000 kg/m^3^. Density of the particle: 1050 kg/m^3^. Diameter of the particle: 5.6 µm. Channel width: 95 µm. Flow rate: 5 µL/h. Applied voltage: 10 V_p-p._

Figure 3 depicts the particle trajectories along the width of the channel for different values of electrode length, and the spacing between two adjacent electrodes. Both the electrode length and spacing are increased equally from 10 µm to 100 µm, keeping all other parameters constant. Moreover, the channel length is kept fixed at 5000 µm in all the cases. It is observed that the displacement of the microparticles along the width at the end of the channel increases initially on increasing electrode length/spacing, until it reaches a maximum value at length/spacing of ~60 µm. Thereafter, the final displacement along the width of the microchannel starts decreasing as the electrode length/gap is further increased. At smaller length/gap, the nDEP force, although stronger in magnitude, is confined to only a smaller effective area above the electrodes. Increasing electrode length/gap reduces the magnitude of the DEP force closer to the electrodes, and increases this area of influence for DEP force, causing the particles to be pushed farther into the microchannel. However, a reverse trend is observed on further increasing electrode length/gap after a certain threshold value. After the optimized gap length, the increased spacing between the electrodes decreases the repulsive nDEP force, causing a decrease in the steady-state deflection along the width of the channel for the particles.

**Figure 4:**
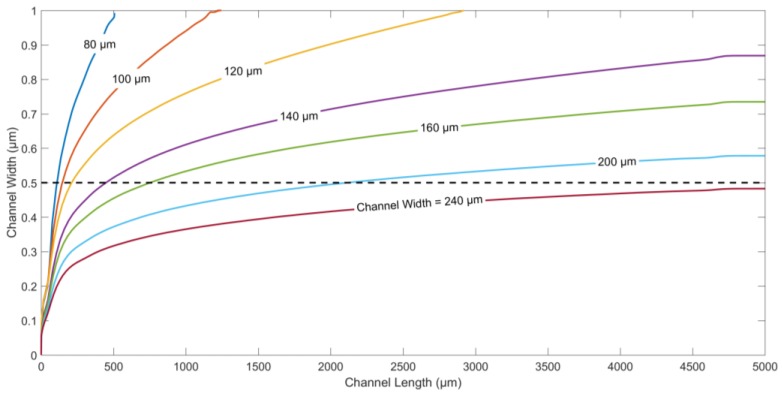
Particles trajectories for different channel width values generated using FEM. Applied frequency: 10 kHz. Medium conductivity: 10 mS/m. Medium density: 1000 kg/m^3^. Density of the particle: 1050 kg/m^3^. Diameter of the particle: 5.6 µm. Electrode/gap width: 60 µm. Flow rate of both fluids: 5 µL/h. Applied voltage: 10 V_p-p._

The particle trajectories for different values of the channel widths and same flow rates of both fluids are displayed in Figure 4. The channel width is changed from 80 µm to 240 µm, to study the effect on the displacement of microparticles along channel width. To ensure dipping or medium exchange in the current device, it is imperative that the particles are pushed beyond the centerline along the width of the channel. For the same electrode/gap width and flow rate, Figure 4 shows that channel widths between 80 µm and 200 µm ensure dipping, and medium exchange operations with different immersion times for the same flow rates of both fluids. The lower the channel width, the faster the target particles are immersed in the second medium. On the other hand, channels wider than 240 µm do not facilitate successful dipping at the selected flow rate, since the nDEP force generated by the electrodes is not enough to push the particles into the second medium beyond the centerline along channel width.

**Figure 5:**
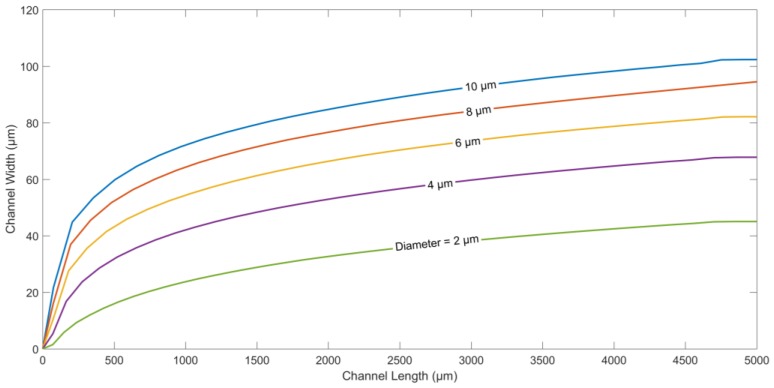
Particle trajectory for different particle diameters. Applied frequency: 10 kHz. Medium conductivity: 10 mS/m. Medium density: 1000 kg/m^3^. Density of the particle: 1050 kg/m^3^. Electrode/gap width: 60 µm. Channel width: 200 µm. Flow rate: 5 µL/h. Applied voltage: 10 V_p-p._

The next parameter studied is the diameter of the microparticles. Figure 5 shows the trajectories of particles with different diameters. DEP force, as indicated in Equation (1), is proportional to cubic radius $r_{p}{}^{3}$ of the particle. On the other hand, the drag force is proportional to only $\;r_{p}$. It is evident that larger the particle size, the larger the DEP force will be, and the higher the displacement in the direction along the width of the channel.

**Figure 6:**
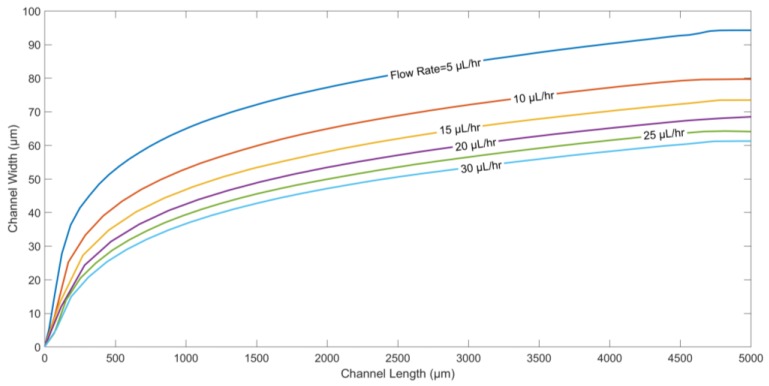
Particle trajectory for different flow rates of fluids in the microchannel. Applied frequency: 10 kHz. Medium conductivity: 10 mS/m. Medium density: 1000 kg/m^3^. Density of the particle: 1050 kg/m^3^. Electrode/gap width: 60 µm. Channel width: 200 µm. Particle diameter: 5.6 µm. Applied voltage: 10 V_p-p_.

In Figure 6, the particle trajectories for different flow rates are generated. The flow rate directly affects the displacement of the particle in the direction along the channel length. Figure 6 suggests that as the flow rate of fluids is decreased, microparticles are pushed deeper into the channel along its width, since the drag force plays a dominant role compared to the other forces. 

**Figure 7:**
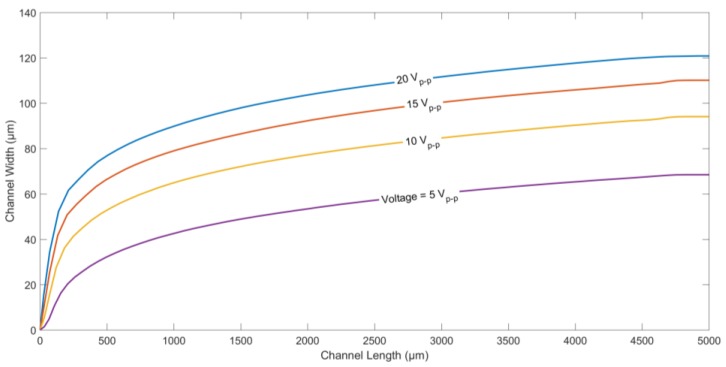
Particle trajectories for different applied voltages. Applied frequency: 10 kHz. Medium conductivity: 10 mS/m. Medium density: 1000 kg/m^3^. Density of the particle: 1050 kg/m^3^. Electrode/gap width: 60 µm. Channel width: 200 µm. Particle diameter: 5.6 µm. Flow rate: 5 µL/h.

Finally, the effect of applied voltage on the trajectory of a microparticle was studied. The particle trajectories for different applied voltages (peak-to-peak) is shown in Figure 7. It can be seen that the higher the applied voltage, the higher the displacement of the microparticles along channel width. Equation (1) shows that the DEP force is proportional to *∇*(*E*·*E*). Hence, higher applied voltages tend to create a higher nDEP force, pushing the microparticles further away from the electrodes.

## 5. Experimental Validation

**Figure 8:**
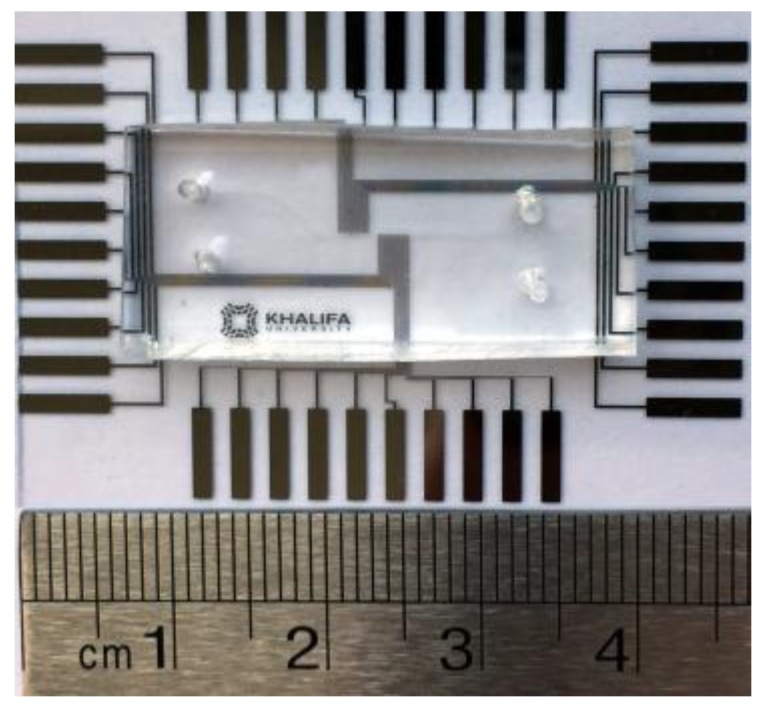
A photograph of the glass wafer containing patterned microelectrodes and the PDMS block. Based on the numerical results, each electrode array is designed to have 10 pairs of electrodes with electrode width, electrodes spacing, and channel width 60 µm, 60 µm, and 80 µm respectively.

The PDMS-based microdevice (Figure 8) to perform the dipping operation successfully is fabricated using standard photolithographic techniques. The microdevice comprises of microelectrodes patterned on a glass wafer bonded with the PDMS channel. The two layers of the device are fabricated separately. A layer of gold (Au) is sputtered on the glass wafer (4 inch diameter and 500 µm thick), after depositing a thin layer of chromium (Cr) on it. The thin Cr layer improves the adhesion of the Au layer on the glass wafer. Next, a positive photoresist Shipley PR1800 (MicroChem Corp., Westborough, MA, USA) is spin-coated, exposed to ultraviolet light using a photolithography system (KLOE 650, KLOE, Montpellier , France), and developed in MF319 developer (MicroChem Corp.), at room temperature, to pattern the photoresist layer on the glass wafer. The exposed Au parts are then etched away, while those protected by the positive photoresist remain unaffected. Next, the Cr exposed layer is etched away using Cr etchant. In the next step, the photoresist is stripped off using acetone, leaving behind the Au microelectrodes on the glass wafer. Finally, the glass wafer is diced so that each piece can be used as a separate device.

In order to make the PDMS microchannels, an SU-8 2025 mold is first patterned on silicon (Si) wafer. Separately, the PDMS base and curing agent are mixed at 10:1 by weight. The mixture is then poured on the mold, degassed, and finally cured at 80 °C for two hours. Both the glass wafer and the PDMS microchannel are cleaned in plasma cleaner, and then aligned under the microscope before they are thermally bonded to each other. A few droplets of ethanol are used to delay the bonding process between the PDMS and glass, and perform the alignment. The microdevice is fabricated using the optimized geometrical parameters—e.g., electrode width, electrodes spacing, channel width, etc.—obtained from numerical analyses.

**Figure 9:**
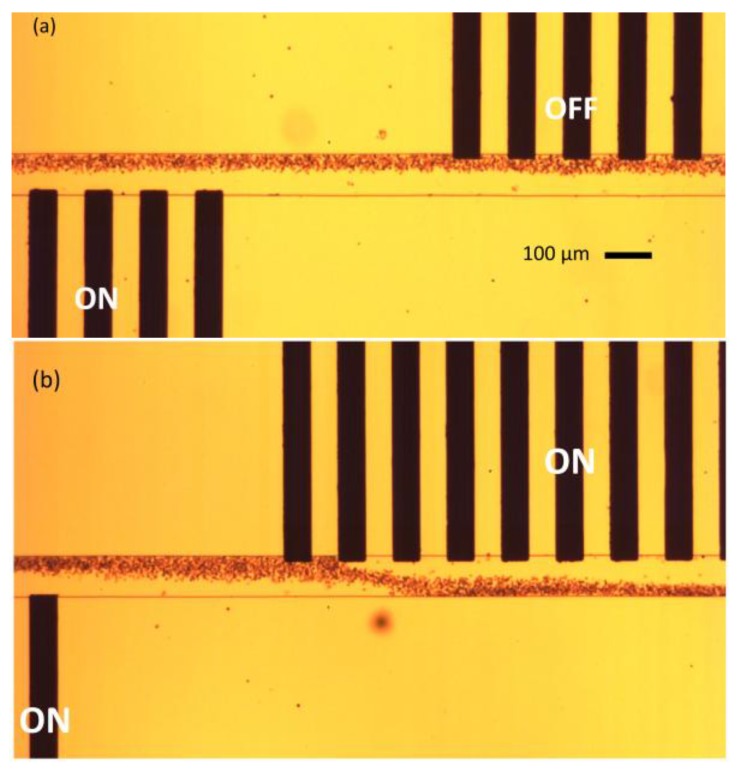
(**a**) The trajectories followed by red blood cells (RBCs) showing medium exchange process. The lower electrodes are powered ON, while the upper electrode sets are powered OFF; (**b**) Operation of cell dipping. Both the electrodes are turned ON. The blood cells are flowing from left to right. Flow rate is 5 µL/h, applied voltage is 20 V_p-p_, applied frequency is 10 KHz, and the frame rate 60 frames per second.

After the device was completed, modified blood samples to carry out experiments was prepared. Human blood samples from anonymous healthy donors were obtained after addressing appropriate institutional ethics and privacy concerns. The participants gave their informed consent for inclusion before they participated in the study. The study protocol was approved on the 19 January 2015 by the Ethics Committee of the office of research support at Khalifa University. The protocol for sample preparation is as follows: (1) The blood sample is first centrifuged in the centrifuging machine to separate the cells from the high conductivity plasma; (2) After being separated from the plasma, the blood cells are placed in a low conductivity sucrose/dextrose medium (10 mS/m) [21]. In the experiments, the cells are allowed to pass through the microdevice using a syringe pump from one of the inlets at a flow rate of 5 µL/h. The medium conductivity of 10 mS/m ensures that RBCs are under the influence of nDEP at the operating frequencies. From the second inlet, only the sucrose/dextrose medium without RBCs with a different conductivity value (12 mS/m) is pumped into the microchannel using a separate syringe pump. An AC signal of 20 V_p-p_ at the frequency of 10 kHz was applied to the electrodes using a function generator. The frequency of 10 kHz is selected to ensure nDEP phenomenon for the blood cells. The effect of the nDEP force on the blood cells is observed under a microscope. Figure 9 shows the results obtained during the experiments. Figure 9a depicts the phenomenon of medium exchange when a single set of electrodes (bottom) is powered ON, while the other (top) set is powered OFF. The blood cells are deflected only once during the medium exchange procedure. Figure 9b explains the phenomenon of blood cells dipping in the microchannel. The cells are initially deflected from the bottom electrode set, and are again deflected from the top electrode set, dipping the cells into the top-flowing medium, and again, to the original bottom medium.

**Figure 10:**
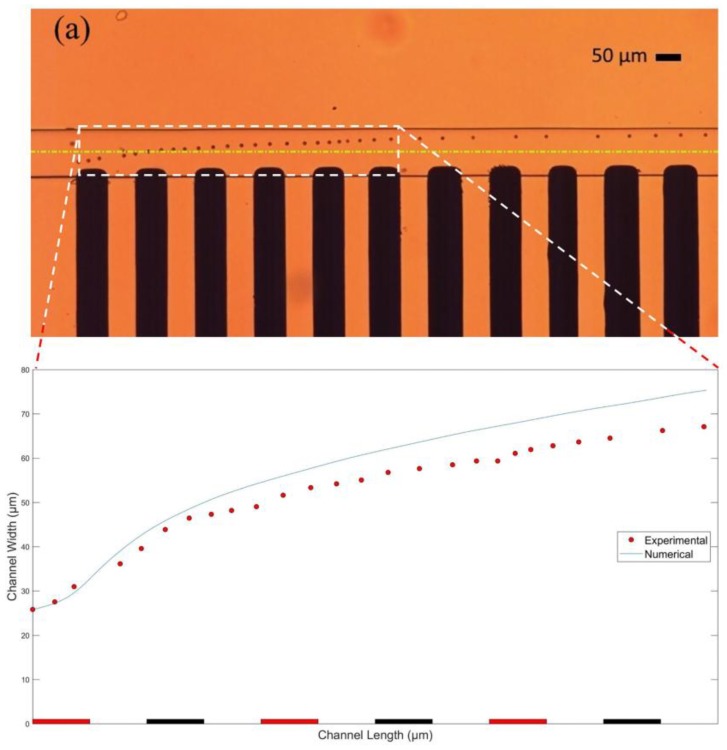
(**a**) Trajectory followed by a single RBC during the experiment; (**b**) Comparison between the experimental and the numerical results with maximum deviation of 12.22%. Electrode width: ~60 µm. Gap between electrodes: ~60 µm. Channel width: ~80 µm. Flow rate: 5 µL/h. Particle diameter: 5.6 µm in FEM and RBC in the experiment. Applied voltage to the electrodes: 20 V_p-p_. Applied frequency: 10 kHz. Frame rate: 60 frames per second.

Separately, the trajectory of a single RBC under the influence of nDEP with one of the electrode sets is also obtained during the experiment. Figure 10 displays a comparison between the path followed by a single blood cell during the experiment, and the trajectory of the cell obtained in the FEM model. A good match can be seen between the experimental and the analytical results. The maximum deviation between the experimental and numerical results, as shown in Figure 10b, is recorded to be only 12.22%.

## 6. Conclusions

In conclusion, a theoretical model of a microfluidic device for dipping and medium exchange processes is presented. Numerical parametric studies are performed for different geometrical and operational parameters. A microfluidic device is fabricated using the optimized parameters obtained in the parametric studies. The experiments involving actual blood cells are performed, and agreement can be seen in both the experimental and numerical trajectories. Finally, the presented model is capable of performing dipping and medium exchange processes in many polarizable cell lines, as long as the applied frequency is below their crossover frequencies to ensure nDEP. Moreover, dipping and medium exchange are possible, as long as the right parameters are selected from the presented parametric study in this paper.
